# Rotavirus-Mediated Suppression of miRNA-192 Family and miRNA-181a Activates Wnt/β-Catenin Signaling Pathway: An In Vitro Study

**DOI:** 10.3390/v14030558

**Published:** 2022-03-09

**Authors:** Anwesha Banerjee, Mamta Chawla-Sarkar, Anupam Mukherjee

**Affiliations:** 1 Division of Virology, ICMR-National AIDS Research Institute, Pune 411026, India; banerjee.anwesha1991@gmail.com; 2 Division of Virology, ICMR-National Institute of Cholera and Enteric Diseases, Kolkata 700010, India; chawlam70@gmail.com

**Keywords:** microRNA, rotavirus, Wnt, β-Catenin, miR-192, miR-181a, antiviral

## Abstract

The significance of the Wnt/β-catenin signaling cascade in Rotavirus (RV) infection has not been elucidated. In this study, we attempt to elucidate the importance of the Wnt/β-catenin pathway in the RV pathogenesis and investigate a miRNA-mediated approach to regulate the pathway to repress the RV infection in the host. The regulation of the Wnt signaling pathway in terms of β-catenin accumulation and activation was analyzed by Western blotting and Confocal imaging analysis. The expression levels of miR-192 family members and miR-181a were enquired into using qPCR assays, whereas their targets in the Wnt pathway were confirmed using the Luciferase Reporter Assays. Members of the miR-192 family and miR-181a, which target the components of the pathway, were also found to be considerably decreased in expression during RV infection. Ectopic expression of these miRNAs could restrict the RV pathogenesis by targeting the intermediates of the Wnt signaling pathway. The miR-192 family and miR-181a were capable of suppressing the RV infection via targeting of the Wnt/β-catenin pathway. The study not only highlights the role of the Wnt signaling cascade in RV infection but also suggests that miRNAs can synergistically decrease RV replication by a significant amount. Thus, the miR-192 family and miR-181a present themselves as prospective antivirals against RV infection.

## 1. Introduction

Before the introduction of the vaccines Rotarix^®^ (RV1) and RotaTeq^®^ (RV5), Rotavirus (RV) was considered to be the primary cause of fatal diarrhea in children below the age of five. The global RV detection rate had decreased from 42.5% in 2000 to about 37% by 2013. Nevertheless, India accounted for about 22% of the global death incidences due to RV, while being one of the four countries that account for about half of the total estimated deaths due to RV infection [1]. Although a four-year study (2016–2019) reported a decrease in Rotavirus-associated gastroenteritis (RVGE) cases with the acceptance of vaccines, most of the sufferers were infants below two years of age, with the most prevalent RV strains being different from those identified in previous studies [2]. Thus, with the change of the epidemiological profile of RVGE in India, it is a tacit interpretation that alternative approaches need to be explored. Belonging to the family Reoviridae, the RV has a 100 nm diameter icosahedral-shaped protein capsid with spikes of VP4. The RV double-stranded RNA genome is segmented and is about 18 Kb. RV has about the same number of structural (VP1-VP4, VP6, and VP7) as well as nonstructural (NSP1-NSP6) proteins [3].

On the other hand, since its discovery 38 years ago, the Wnt/β-catenin pathway has been associated with cell survival and proliferation. The Wnt family contains about 19 glycoproteins capable of binding to the members of the Frizzled receptor family of G-protein coupled receptors. This receptor–ligand binding disrupts the destruction complex for β-catenin by restraining the components of the destruction complex at the cell membrane, leading to the accumulation and activation of β-catenin for cell survival. Without the ligand binding interaction, the β-catenin is anchored by the destruction complex comprising of the Axin, APC, and Glycogen synthase kinase 3 beta (GSK3β). The destruction complex component, GSK3β, phosphorylates and destabilizes the β-catenin at Ser33, Ser37, and Thr41, followed by proteasomal degradation of the catenin protein. The accumulated β-catenin localizes into the nucleus, where, along with TCF/LEF, it transactivates the gene expression of Cyclin D, c-Myc, and CD44 to regulate cell survival and proliferation. Therefore, the Wnt/β-catenin pathway is a popular target in anti-cancer therapeutics [4,5]. The manipulation of the Wnt/β-catenin pathway by microRNAs (miRNAs) is a well-established concept. The miRNAs are short RNA sequences that do not themselves code for protein but regulate the expression of many protein-coding genes. They are the evolutionarily conserved ~22 nucleotides gene regulators capable of targeting more than one gene (500 on an average) for suppression [5]. Thus, by modulating the miRNAs, it is possible to impart a check on the cell signaling cascade. This study makes an effort to emphasize the significant participation of the Wnt/β-catenin pathway in RV pathogenesis while highlighting the miRNAs that may be targeted to regulate the Wnt/β-catenin pathway as an approach to restrict RV pathogenesis. 

## 2. Materials and Methods

### 2.1. Cell Lines and Virus Strains

Two epithelial cell lines, namely, Caco-2 and MA104, one from the human colorectal adenocarcinoma and another from the African Green Monkey kidney, respectively, and the highly transfectable derivative of the human embryonic kidney cells (293T) were cultured and maintained in 10–20% heat-inactivated fetal bovine serum (FBS), 2 mM sodium pyruvate, 0.1 mM non-essential amino acids (NEAA), and 1% antibiotics supplemented minimum essential medium (MEM) or Eagle’s minimum essential medium (EMEM) in a 5% CO_2_, 37 °C humified incubator. The cell-culture-adapted human RV strains Wa, Ku, and Simian RV strain SA11 (H96) were used for this study. These strains were cultivated and propagated in the MA104 cells, extracted, and titrated via the plaque assay to establish the viral titer [6,7]. RV activation was achieved by exposure to 10 g/mL acetylated Trypsin at 37 °C prior to infection followed by infection of the cells with 3MOI (multiplicity of infection) of the pre-activated virus, at 37 °C for 45 min. All the protocols concerning cell culture, virus propagation, and virus infection were performed under the Biosafety Level (BSL)-2 conditions.

### 2.2. miRNA/siRNA Cytotoxicity Assay

The cytotoxic effect of the transfected miRNA/siRNA was checked. Cells were transfected with scrambled miRNA or siRNA-Fz9 or mimics of miR-192/215 and/or miR-181a in 96-well plates. As per the manufacturer’s protocol (Promega, Madison, WI, USA: G3581), after the transfection procedure, the reagent solution was added onto the cells in a serum-free medium and maintained at 32 °C, 5% CO_2_. A spectrophotometric reading at 490 nm gave a measure of the cell viability. The control/ mock cells were considered to have a 100% viability, and the percentage viability in the transfected cells was calculated in comparison to the mock cells. 

### 2.3. Reverse Transcription and Quantitative PCR

The RNA extraction method using the Trizol reagent (Invitrogen, Carlsbad, CA, USA: 15596018) for the isolation of total RNA was carried out, as instructed by the manufacturer. The miR-192/215, miR-181a, and U6- specific primers were used for the cDNA preparations via reverse transcription of the specific miRNAs using the TaqMan miRNA reverse transcription kit (Applied Biosystem, Pleasanton, CA: 4366597). The cDNA for the RV-VP6 and GAPDH mRNAs was prepared via reverse transcription using the SuperScript™ III First-Strand Synthesis System (Invitrogen, Carlsbad, CA, USA: 18080-051) with random hexamers. For the quantitative real-time PCR for miRNA expression, the TaqMan universal PCR master mix (Applied Biosystem, Carlsbad, CA, USA: 4304437) was used to perform the specific quantification of hsa-miR-192 (Assay ID: 000491); hsa-miR-215 (Assay ID: 000518); hsa-miR-181a (Assay ID: 000480); and the endogenous miRNA control U6 (human origin) (Assay ID: 001973). Specific primers designed to determine viral gene expression of the RV-VP6 gene and the endogenous control GAPDH were used with the SYBR Green Master mix (Applied Biosystems, Carlsbad, CA, USA: 4367659). The formula, 2^−ΔΔCt^ was used for the determination of the relative miRNA and viral gene expression levels after normalization with their respective endogenous controls. 

### 2.4. Cloning and Transfection

The targets of miR-192, miR-215, and miR-181a were predicted using miRWalk and TargetScan. The 3′UTR of the Fz9; SMAD4; TCF7; CCND2; and CTNNB1 were PCR-amplified and digested by the MluI and HindIII restriction enzymes. The pMIR-REPORT miRNA expression luciferase reporter plasmid vector (Ambion Carlsbad, CA, USA: AM5795) was also digested by the same restriction enzymes so that the PCR-amplified and restriction-digested 3′UTR of the targets were cloned at the MluI/HindIII site of the vector. The 293T cells were transfected/co-transfected with the pMIR-REPORT plasmid vector having the 3′UTR of the predicted targets at the cloning site and/or the mimics of hsa-miR-192, miR-215, miR-181a, siRNA-Fz9, or the scrambled miRNA, or the scrambled siRNA at a final concentration of 10–40 nM with Lipofectamine 2000 (Invitrogen, Carlsbad, CA, USA: 11668019), as per the manufacturer’s protocol. 

### 2.5. Luciferase Reporter Assay

The cloned 3′UTR of the miRNA targets contained in the pMIR-REPORT was co-transfected with the mimics of miR-192, miR-215, miR-181a, or scrambled miRNA in order to validate the miRNA targets in terms of measurements of their relative luciferase activities. The relative luciferase activities in the presence or absence of the miRNA were measured by the Dual Luciferase Assay System (Promega, Madison, WI, USA: E1960) post-normalization with the Renilla Luciferase Expression.

### 2.6. Immunoblotting

After the suitable endpoints were achieved in the experiments, the cells that were washed with phosphate-buffered saline (PBS) were then lysed in RIPA buffer. The protein concentrations in the cell lysates were quantified by the PierceTM BCA Protein Assay Kit (Thermo Fisher Scientific, Waltham, MA, USA: 23225). A polyacrylamide gel electrophoresis followed by transferring the proteins onto the PVDF membrane marked the first step in the immunoblotting procedure. 5% non-fat containing milk was used to block the untransferred regions on the membranes. The membranes were then probed with antibodies specific to the proteins phospho-β-catenin ser33/37 (Santa Cruz Biotechnology, Santa Cruz, CA, USA: 57535); phospho-β-catenin ser552 (Cell Signaling Technology, Danvers, MA, USA: 5651); β-catenin (Cell Signaling Technology, Danvers, MA, USA: 8480); ICAT (Santa Cruz Biotechnology: 293489); SMAD4 (Abcam, Waltham, MA, USA: 110175); TCF1/7 (Cell Signaling Technology, Danvers, MA, USA: 2203); CCND2 (Santa Cruz Biotechnology: 56305); Wnt-1 (Santa Cruz Biotechnology: 514531); Wnt-5a (Santa Cruz Biotechnology: 365370); phospho-LRP6 (Cell Signaling Technology, Danvers, MA, USA: 2568); LRP6 (Cell Signaling Technology, Danvers, MA, USA: 3395); phospho-Axin (Merck Millipore, Burlington, MA, USA: ABN1032); Axin (Santa Cruz Biotechnology: 293190); Naked-1 (Cell Signaling Technology, Danvers, MA, USA: 2201), RV-NSP1 (a kind gift from Prof. Koki Taniguchi); RV-NSP4 (prepared according to the standard protocol from Department of Virology and Parasitology, Fujita Health University School of Medicine, Aichi, Japan); and RV- VP6 (HyTest, Turku, Finland: 3C10). These antibodies were either itself tagged with horseradish peroxidase (HRP) or detected via secondary antibodies tagged to HRP, followed by recording of chemiluminescence after the addition of ECL substrate (Millipore, Burlington, MA, USA) in the Chemidoc imaging system (BioRad, Hercules, CA, USA) or developed onto the BioMax Films (Kodak, Rochester, NY, USA). All the PVDF membranes were stripped and reprobed with the internal loading control GAPDH (Santa Cruz Biotechnology: 47724 HRP). The ImageJ software v1.53a (NIH, Bethesda, MD, USA) was used to measure the band intensities followed by normalization with GAPDH and represented in terms of relative fold changes. The immunoblots displayed in this manuscript were compliant with the digital image and followed the integrity policies.

### 2.7. Immunofluorescence

Caco-2 and MA104 cells were cultured on glass coverslips and incubated after infection with RV-SA11 for 6 and 12 h. These cells were then fixed with 4% *w*/*v* paraformaldehyde in PBS and processed for the immunofluorescence technique, as mentioned previously [8]. Cells were incubated with the antibody dilutions specific to RV-VP6 and β-catenin (1:100) at 4 °C overnight, followed by incubation with FITC- (green) and Rhodamine- (red) conjugated secondary antibodies, respectively, in the dark for 2 h in a 37 °C incubator. The nuclei in the cells were stained with Vectashield containing 4′,6′-diamidino-2-phenylindole (DAPI-blue) and visualized with the help of the Axioplan confocal microscope (Carl Zeiss, Jena, Germany). The ZEN Blue software v3.1 (Carl Zeiss Microscopy, Jena, Germany) was used to process the microscopic images stored in the RGB format. In order to compare different images appropriately, these images were captured under identical excitation and detection settings in one session.

### 2.8. Statistical Analyses

The mean of at least two independent experiments plus the standard deviations were calculated and represented as the results of this study in this manuscript. The *p*-value < 0.05, which is considered to be statistically significant, was observed in all the experiments and represented with asterisks. The number of asterisks indicate the significance levels (* *p* < 0.05, ** *p* < 0.01 and *** *p* < 0.001). The significance levels have been analyzed via the Student’s *t*-test. GraphPad Prism 8.4.3 (San Diego, CA, USA) was used to analyze those figures having more than two experimental groups. 

## 3. Results

### 3.1. RV Infection Triggers β-Catenin Accumulation

**Figure 1:**
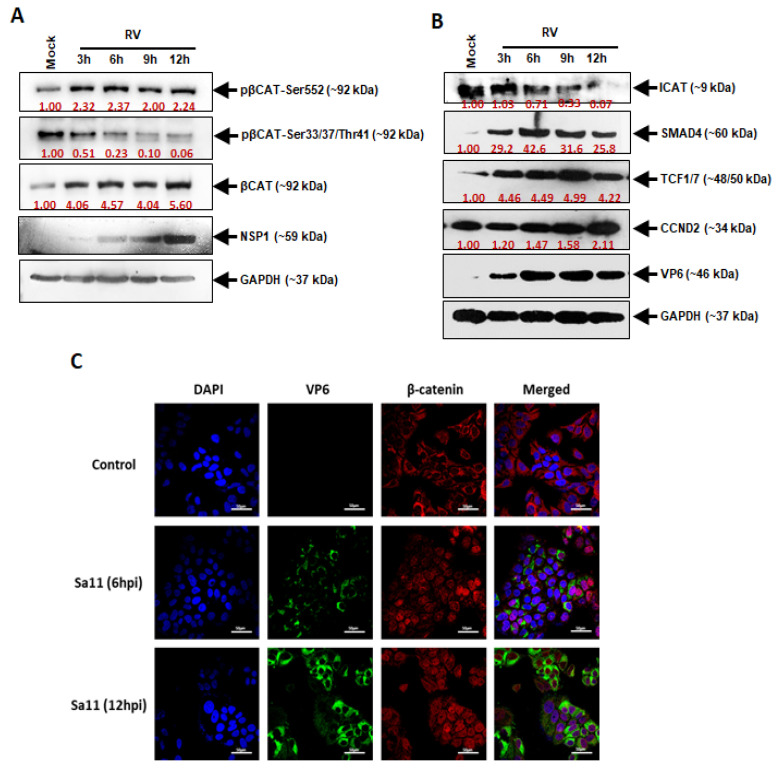
RV infection triggers β-Catenin accumulation. Cells infected with RV were harvested at different time points followed by protein expression analysis by Western blotting and Confocal microscopy techniques. (**A**) RV upregulates β-catenin expression during early (3–12 h) hours of infection. Expression of phospho β-catenin Ser37 downregulated in RV infected cells. Expression of phospho β-catenin Ser552 upregulated in RV infected cells; (**B**) Changes in the expression levels of different regulators of the Wnt/β-catenin cellular signaling pathway. Expression of ICAT downregulated in RV infected cells. SMAD4 is activated during RV infection and acts as an attenuator of β-catenin proteasomal degradation. The downstream molecule of β-catenin and SMAD4, TCF1/7 and CCND2 are also activated during RV infection; (**C**) Phosphorylation at Ser552 and destabilization of Ser37 induces β-catenin accumulation in the cytoplasm and nucleus as evident from IF imaging. Scale bars: 50 μm.

In order to understand the regulation of cell cycle progression by RV, the effect of RV infection on β-catenin mediated cell cycle control was investigated. The accumulation of β-catenin in the cells was observed in the early hours, i.e., 3 h to 12 h post-RV infection. The β-catenin that phosphorylated at Ser-552 was observed to increase with the progression of infection, whereas phosphorylation at Ser-33/37/Thr-41 decreased as the infection progressed. Decreased phosphorylation at specific sites, namely, Ser-33/37/Thr-41, showed that the level of activated β-catenin was maintained at 12 h post-infection and indicated that RV infection was capable of inducing the β-catenin signaling pathway in Caco2 cells (Figure 1A). In addition, ICAT, an inhibitor of the β-catenin pathway, showed reciprocal expression levels as compared to β-catenin. Meanwhile, SMAD4, an activator of the Wnt signaling pathway, showed elevated expression levels up to 12 h after RV infection. TCF1/7 and CCND2, the downstream intermediates of the β-catenin pathway were also elevated, indicative of induction of the β-catenin pathway (Figure 1B). Besides, immunofluorescence imaging of the RV-SA11-infected Caco2 cells illustrated the accumulation of β-catenin in the cytoplasm and in the nucleus upon advancement of infection up to 12 h (Figure 1C). Together, it could be inferred that RV infection triggers the onset of the β-catenin signaling pathway with β-catenin accumulation in the cytoplasm and nucleus during the early hours of infection. 

### 3.2. Wnt-1/Wnt-5a Pathway Activates during RV Infection

**Figure 2:**
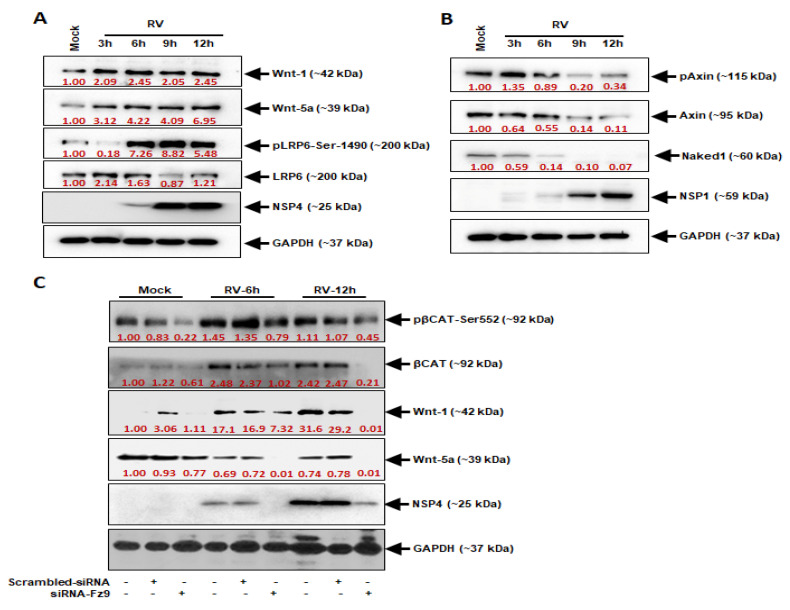
Wnt-1/Wnt-5a pathway activates during RV infection. (**A**) Expression of Wnt-1 and Wnt-5a have been gradually upregulated during RV infection. LRP6 has also been hyper phosphorylated during RV infection; (**B**) Phosphorylation of Axin and its total component have been reduced in RV infected cells. Expression of Nkd1 is suppressed in RV infected cells; (**C**) Phosphorylation of β-catenin Ser552 significantly inhibited in siRNA-FzD9 treated but RV infected cells. The total β-catenin level is also suppressed. The expressions of Wnt-1 and Wnt-5a are also inhibited in siRNA-FzD9 treated but RV infected cells. This may be the result of negative feedback mechanism of Nkd1 or Axin or may be that some other factors are involved in this pathway. The siRNA-FzD9 restricted the RV infection too, as evident by RV-NSP4 expression.

The accumulation of β-catenin is possible only when it has escaped the destruction complex, which is regulated by Wnt. Therefore, it is acceptable that this accumulation is encouraged by the induction of Wnt signaling. We checked the protein expression levels of Wnt-1 and Wnt-5a, which showed a similar gradual elevation pattern as that of β-catenin. LDL receptor-related protein 6 (LRP6), a co-receptor of Wnt, also exhibited increased phosphorylation at Ser-1490, which was maintained up to 12 h post-RV infection, indicating that the Wnt pathway had been triggered (Figure 2A). On the other hand, the de-regulators of the Wnt pathway, namely, Naked-1 (Nkd-1) and phosphorylated Axin (pAxin) were reciprocally expressed to Wnt and LRP6 phosphorylation after 12 h of infection (Figure 2B). As the Frizzled receptors are one of the initiator proteins of the Wnt signaling, the involvement of the Wnt/β-catenin signaling in RV infection was confirmed by suppressing Frizzled-9 (Fz9) with small-interfering RNAs specific to Fz9 (siRNA-Fz9) (Figure 2C). The siRNA-Fz9 could indirectly suppress the phosphorylation of β-catenin at Ser-552 as well as the total β-catenin levels, both in the mock as well infected samples. Wnt-1 and Wnt-5a were also suppressed as a result of siRNA-Fz9 transfection. Moreover, the inhibition of Fz9 expression by siRNA successfully reduced the protein expression levels of RV-NSP4, which is directly suggestive of reduction in RV infection (Figure 2C). Such a reduction was not observed in the case of transfection with a scrambled-siRNA or RV-infection without siRNA transfection, thus expressly signifying that the Wnt-1/Wnt-5a signaling pathway is activated during the early hours of RV infection. This confirmation encouraged us to look at the Wnt pathway intermediates as therapeutic targets to alleviate the RV infection in the host.

### 3.3. miR-192 Family and miR-181a Targets Key Regulators of Wnt/β-Catenin Signaling Pathway

**Figure 3:**
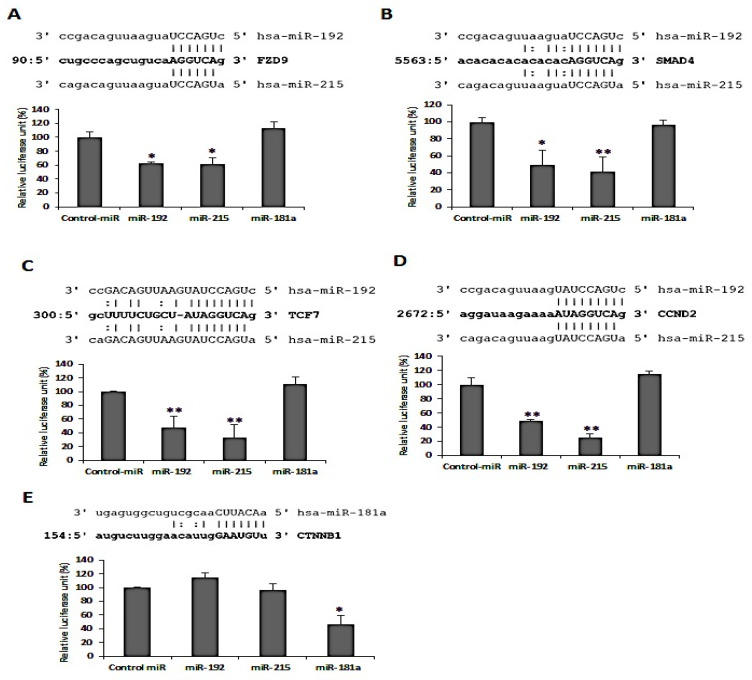
miR-192 family and miR-181a target key regulators of Wnt/β-Catenin signaling pathway. Cloning of the 3′UTR of some of the intermediates of the Wnt pathway followed by Luciferase reporter assay revealed that (**A**–**D**) FZD9, SMAD4, TCF7 andCCND2 are the direct targets of miR-192 andmiR-215; whereas, (**E**) miR-181a is responsible for targeting CTNNB1 or β-catenin itself. * *p* < 0.05, ** *p* < 0.01.

The involvement of the Wnt/β-catenin pathway in RV infection involves a number of miRNAs in RV replication within the epithelial cells. In addition, several miRNAs have their targets involved in the Wnt/β-catenin pathway. Thus, the idea of RV infection manipulating the cellular miRNAs to modulate the Wnt/β-catenin pathway was perceived. The Frizzled receptors (Fz9/FZD9) were comprehended to be the target of two-member miRNAs of the miR-192 family, miR-192, and miR-215, clearly depicting around 35% decreased luciferase activity as compared to the control (Figure 3A). Similarly, SMAD4, TCF7, and CCND2 were found to be the direct targets of hsa-miR-192 and -miR-215 with an inhibition of 50–70% (Figure 3B–D). The β-catenin (CTNNB1), however, is the direct target of the has-miR-181a with a 50% inhibition of β-catenin expression (Figure 3E), while no such inhibition was observed with miR-181a transfection in the cases of FZD, SMAD4, TCF7, or CCND2. In all, the miRNAs that directly target the signaling components of the Wnt/β-catenin pathway were confirmed.

### 3.4. RV Infection Downregulates the Expressions of miR-192, miR-215, and miR-181a

**Figure 4:**
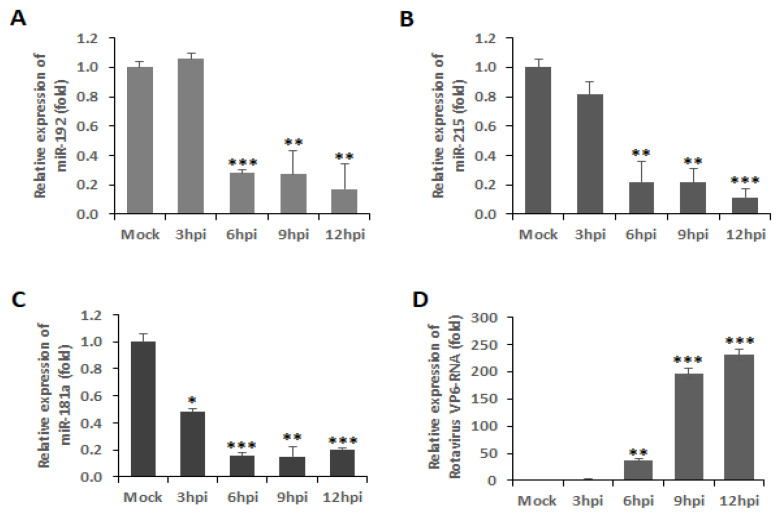
RV infection downregulates the expressions of miR-192, miR-215, and miR-181a. cDNA preparations from RNA isolated from 3–12 h. RV infected cells were checked for miRNA expression using qPCR. (**A**–**C**) Expressions of miR-192, miR-215 and miR-181a were downregulated in RV-infected MA104 cells; (**D**) Level of viral RNA (RV-VP6) was also checked in the same lysates to confirm RV infection. * *p* < 0.05, ** *p* < 0.01 and *** *p* < 0.001.

After identifying the miRNAs that could interfere with the expressions of their targets in the Wnt pathway, we then looked for changes in the expression levels of these respective miRNAs in RV infection. Real-time PCR results disclosed that the expression levels of miR-192, miR-215, and miR-181a decreased with an increase in RV infection of MA104 (data not shown) and Caco2 cells (Figure 4). Where the mRNA levels of VP6, an intermediate capsid structural protein of RV, continued to rise till 12 h post-infection (Figure 4D), the expression of miR-192 decreased by about 80% compared to uninfected mock samples (Figure 4A). The miR-215 expression was downregulated by about 90% (Figure 4B), and the expression of miR-181a declined around 70%, at 12 h post-RV infection (Figure 4C). Therefore, it could be inferred that RV prompts the Wnt/β-catenin signaling pathway by downregulating the miRNAs, which target the significant components of the pathway.

### 3.5. miR-181a and miR-192/215 Inhibits RV Replication

**Figure 5:**
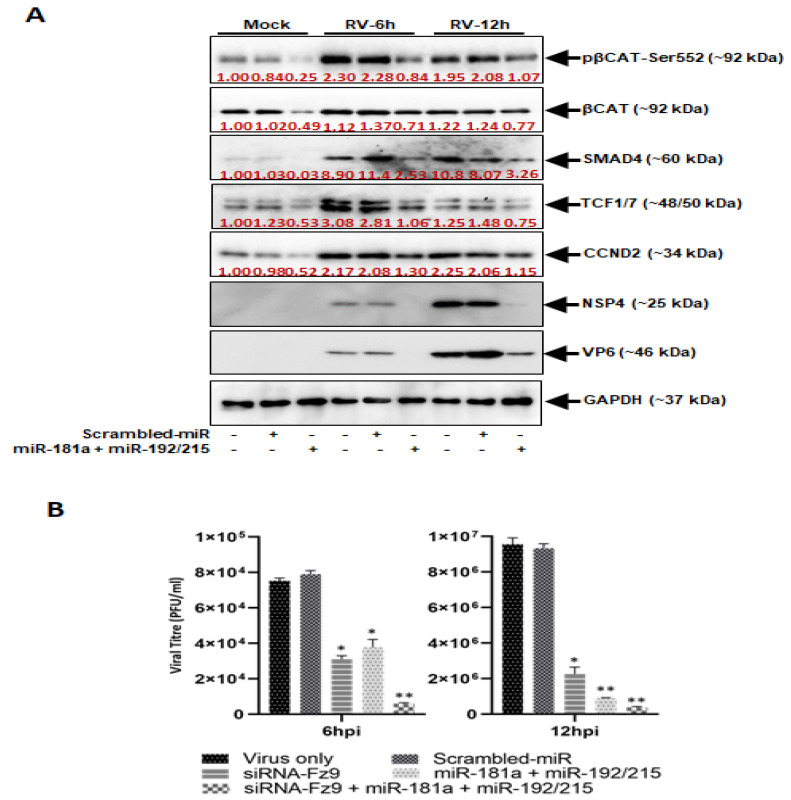
miR-181a and miR-192/215 inhibit RV replication. (**A**) Overexpression of miR-181a in presence of miR-192 andmiR-215 suppressed the phosphorylation of β-catenin in RV-infected MA104 cells. Expression of SMAD4, TCF1/7, and CCND2 is downregulated in presence of miR-192 family miRNA and miR-181a. Expression of RV structural protein VP6 and nonstructural protein NSP4 were also suppressed in presence of mimic miR-181a and miR-192/215; (**B**) Viral titer was measured at 6 hpi and 12 hpi in siRNA-Fz9 transfected and miR-181a and miR-192/215 overexpressed MA104 cells. RV replication was significantly suppressed in presence of either siRNA-Fz9 or mimic miR-181a and miR-192/215. The combined effect of siRNA-Fz9, miR-181a and miR-192 mimics creates much more significant anti-rotaviral state. * *p* < 0.05, ** *p* < 0.01.

Confirmation of the participation of miRNAs in the RV-mediated upregulation of the Wnt/β-catenin pathway led us to investigate its role in assuaging the RV infection. The role of miR-192/215 and miR-181a was confirmed by using mimics of these miRNAs to check the modulations in the Wnt/β-catenin pathway as well as the changes in the virus titer (Figure 5). Overexpression of miR-192/215 and miR-181a in the RV-infected Caco2 cells revealed that the combined effect of miR-192/215 and miR-181a could suppress the phosphorylation of β-catenin at Ser-552, 6 h and 12 h post-RV infection. Similarly, the other signaling intermediates of the pathway, SMAD4 and TCF1/7, and the one directly involved in the cell cycle control, CCND2, showed decreased expressions compared to RV infected and miRNA transfected samples. The expressions of the RV viral proteins, VP6 (structural protein) and NSP4 (nonstructural protein), were also reduced in the RV infected-miR-192/215-miR-181a overexpressed samples (Figure 5A). In addition, the virus titer, a direct indication of the severity of virus infection, showed ~2-fold decrease as compared to only infected controls at 6 h, and ~10 fold at 12 h post-RV infection. Moreover, the combined effect of miR-192/215-miR-181a and siRNA-Fz9 could decrease the virus titer to ~20 folds, thus successfully making the RV infection plummet in the Caco2 cells (Figure 5B).

## 4. Discussion

Inhibition of cell cycle arrest is a common mechanism adopted by oncoviruses leading to cancer establishment in the host. Although RV has not been established as an oncovirus, our previous study has reported the involvement of RV in the manipulation of the cancer-associated epithelial-mesenchymal transition (EMT) pathway for efficient viral spread [9]. Apart from this, there have been other reports emphasizing the significance of cell cycle progression in RV pathogenesis [7,10,11]. Similarly, our findings support these previous reports via evidence proving the induction of the Wnt/β-catenin signaling pathway during the early hours of RV infection. The initiation of the Wnt/β-catenin pathway is marked by the accumulation and activation of β-catenin in the cytoplasm followed by its translocation to the nucleus (Figure 1), where it acts as a transcriptional coactivator of cell survival and proliferation-associated genes of Cyclin D1 (CCND1), MYC, etc. [12]. The sustained activation of β-catenin was shown to be maintained by the increased phosphorylation at Ser552 and decreased phosphorylation at Ser37 (Figure 1). This is in compliance with the reports of increased activation of Akt and PKA during RV infection [7], both of which are Serine/Threonine kinases phosphorylating β-catenin at Ser552 to activate it [13,14]. In support of this, SMAD4, which is an activator of the β-catenin pathway, increases in the same pattern as that of β-catenin itself, whereas ICAT, an inhibitor of the pathway, is reduced in expression (Figure 1). This is because SMAD4 is a potent inducer of the Frizzled-4 receptor of the Wnt signaling, ultimately leading to the activation of β-catenin [15]. ICAT, on the other hand, disrupts the interaction of β-catenin with the transcription factor TCF4 to inhibit the β-catenin pathway [16]. Since the activation of β-catenin is regulated by the interaction of the Wnt proteins with the GPCRs, an investigation of the Wnt protein levels revealed an increase in protein production similar to that of active β-catenin protein levels (Figure 2). Specifically, Wnt is the regulator of a ‘destruction complex’, which the β-catenin escapes in order to contribute to the cell cycle progression [4]. The low-density lipoprotein receptor-related protein 6 (LRP6), a co-receptor of Wnt, is also activated with phosphorylation at Ser1490, which is maintained up to 12 h (Figure 2). This phosphorylation of LRP6 is dependent on a scaffold protein, Dishevelled (Dvl). In fact, a signalosome complex induced by Wnt and comprising co-clustered receptors, Dvl and LRP6, is a prerequisite for LRP6 phosphorylation, ultimately leading to the stabilization of β-catenin [17]. Expression of the negative regulators of Wnt, Axin, and Naked (Nkd) were also found to be downregulated at 12 hpi in our study, so as to infer that the Wnt pathway is not only induced upon RV infection but is also maintained during the 12 early hours of infection (Figure 2). Moreover, Axin phosphorylation, which is the guardian of the destruction complex and is thus upheld during the basal conditions, is downregulated, indicating that the Wnt pathway has been triggered upon RV infection [18]. Naked Cuticle Homolog-1 (Nkd-1) also deregulates the Wnt pathway by interacting with β-catenin and preventing its accumulation in the nucleus [19]. Where the Wnts are the ligands initiating the β-catenin pathway, Frizzled (FzD) proteins are the GPCRs to the ligands and are equally essential in the activation of the pathway [20]. Therefore, siRNA, specifically inhibiting the FzD-9 function, confirmed the involvement of the Wnt/β-catenin pathway in RV infection by reducing the expression of Wnt-1, -5a, decreasing activation phosphorylation on Ser552 residue of β-catenin, as well as ultimately decreasing the expression of the RV viral protein NSP4 (Figure 2). Thus, FzD-9 could be considered a probable therapeutic target for reducing RV pathogenesis. Interestingly, the miRNAs of the 192 family, miR-192 and miR-215, could establish themselves as effective suppressors of more than one crucial intermediate in the β-catenin pathway, namely, FzD-9, SMAD4, TCF7, and CCND2. Another miRNA, miR-181a from the miR-181 family of miRNAs, targets β-catenin itself (Figure 3), thus suggesting that a combinatorial effect of these miRNAs may be able to inhibit the exploitation of the β-catenin pathway by RV to reduce the RV pathogenesis. Since RV infection brought about a 70–90% decrease in the expression of the miRNAs 192, 215, and 181a (Figure 4), the combined overexpression of these miRNAs could not only retard the β-catenin signaling cascade but also reduced the expression of the RV proteins, VP6 and NSP4. Additionally, where the combined overexpression of these miRNAs could reduce the RV viral titer to about 10 folds at 12 hpi, transfection of siRNA-Fz9 along with the mimics of these miRNAs could reduce the viral titer to about 20 folds (Figure 5). Our study establishes that the Wnt/ β-catenin pathway is triggered and exploited by RV infection. The exploitation of the Wnt/ β-catenin pathway has been reported in other infections as well, be it bacterial infections such as Salmonella infection and tuberculosis, protozoan such *Trypanosoma cruzi* infection, or viral infections such as HIV [21]. As established in our previous studies, cell survival and proliferation are an imperative event for RV spread and pathogenesis, and involves a number of mechanisms to do so, such as manipulation of the Ca^2+^/Calmodulin pathway, the epithelial-mesenchymal transition pathway, and suppression of apoptosis [7,9,11]. This study supports the evidences of our previous findings that RV allows the host target cells to survive and propagate so that it may spread to other uninfected cells and build its pathogenesis in the host. Our study goes forward to identify the miRNAs that may serve as potential therapeutic candidates against RV infection. These miRNAs are well associated with other viral scenarios such as hepatitis C, hepatitis B, influenza, tuberculosis, etc. [22,23,24,25,26,27,28], but our study is the first report of them being associated in RV infection.

## 5. Conclusions

**Figure 6:**
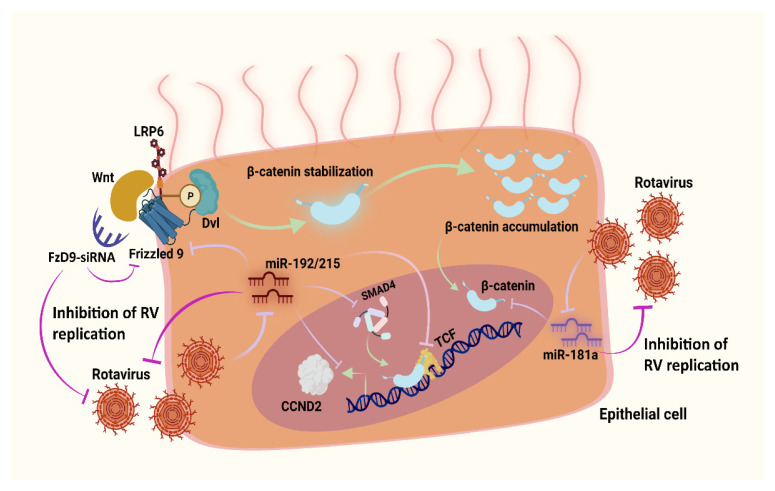
Schematic representation of the RV-induced changes in the Wnt/β-catenin signaling and the role of miR-192/215, miR-181a and siRNA-FzD9 in the establishment of an anti-rotaviral state. RV triggers the activation of the Wnt/β-catenin signaling pathway in the epithelial cells by negatively regulating the miRNAs 192, 215 and 181a. Ectopic expression of these miRNAs along with an siRNA for the receptor of the Wnt (FzD9) can significantly suppress the Wnt pathway, ultimately leading to the decrease in RV replication.

In summary, this study highlights the involvement of the cell survival associated pathway Wnt/β-catenin in RV pathogenesis and substantiates the role of prospective miRNA therapeutic agents that target the components of the pathway to extenuate RV pathogenesis and spread (Figure 6). This, along with our previous reports, conceptualizes that, by interrupting the spread of RV pathogenesis via the combined administration of these miRNAs, the virus titer in the host can be controlled to a considerable extent. 

## Data Availability

Not applicable.
